# Improvements in health-related quality of life with esketamine nasal spray versus quetiapine extended release

**DOI:** 10.1192/j.eurpsy.2025.10123

**Published:** 2025-10-14

**Authors:** Andreas Reif, Bernhard T. Baune, Jozefien Buyze, Anthony J. Cleare, Shaun Johnson, Yerkebulan Kambarov, Nigel Olisa, Falk Schuster, Christian von Holt, Tamara Werner-Kiechle, Eduard Vieta

**Affiliations:** 1 Department of Psychiatry, Psychosomatic Medicine and Psychotherapy, University Medical Centre Frankfurt, Frankfurt am Main, Germany; 2 Fraunhofer Institute for Translational Medicine and Pharmacology ITMP, Frankfurt am Main, Germany; 3 Department of Psychiatry, University of Münster, Münster, Germany; 4 Department of Psychiatry, The University of Melbourne, Melbourne, Australia; 5 Johnson & Johnson, Beerse, Belgium; 6 Institute of Psychiatry, Psychology & Neuroscience, King’s College London, London, UK; 7 GAMIAN-Europe, Brussels, Belgium; 8 Independent Consultant, Leipzig, Germany; 9 Johnson & Johnson, Neuss, Germany; 10 Institute of Neuroscience, University of Barcelona, Hospital Clinic, IDIBAPS, CIBERSAM, Barcelona, Spain

**Keywords:** clinical trial, esketamine, health-related quality of life, quetiapine, treatment-resistant depression

## Abstract

**Background:**

Clinical response and remission may not fully reflect patient priorities in treatment-resistant depression (TRD). Health-related quality-of-life (HRQoL) outcomes should be assessed to comprehensively capture treatment benefits.

**Methods:**

ESCAPE-TRD (NCT04338321) was a 32-week randomized, phase IIIb trial comparing esketamine nasal spray (NS) versus quetiapine extended release (XR), both alongside an ongoing selective serotonin reuptake inhibitor/serotonin-norepinephrine reuptake inhibitor, in patients with TRD. Symptom and HRQoL improvements were assessed using the Patient Health Questionnaire-9 (PHQ-9), 36-Item Short Form Survey (SF-36), Quality of Life in Depression Scale (QLDS), and EuroQoL 5-Dimension 5-Level (EQ-5D-5L) measures.

**Results:**

Esketamine NS-treated patients (*N*=336) reached PHQ-9 remission (score ≤4) quicker than quetiapine XR-treated patients (*N*=340), and more had remission by Week 32 (34.5% vs. 18.2%; odds ratio [OR]: 2.39 [1.67, 3.41], *p*<0.0001). “Role Emotional”, “Mental Health”, and “Social Functioning” SF-36 domains showed significantly greater improvements in esketamine NS-treated patients compared with quetiapine XR-treated patients at Week 32 (*p*<0.05), returning to levels close to general population norms. More esketamine NS-treated patients had a meaningful improvement in their QLDS score by Week 32 (60.7% vs. 41.8%; OR: 2.16 [1.59, 2.94], *p*<0.0001), and reached this improvement quicker than quetiapine XR-treated patients. Proportions of patients reporting an EQ-5D-5L score of 1 (no problems) were significantly higher across all domains with esketamine NS versus quetiapine XR at Week 32 (*p*<0.05).

**Conclusions:**

Esketamine NS produced superior improvements in HRQoL compared with quetiapine XR, indicating positive impacts on aspects of patients’ lives that matter to them, alongside clinical symptoms of TRD.

## Introduction

According to the World Health Organization (WHO), depressive disorders are the largest contributor to loss of healthy life globally [1]. The high prevalence of major depressive disorder (MDD) leads to substantial negative impacts on patients’ daily lives, cognitive function, and the ability to perform and enjoy occupational and social activities [2]. As a result, the health-related quality of life (HRQoL) of patients with MDD is significantly lower than that of individuals with chronic medical disorders, such as hypertension, cancer, or chronic pain [3].

Between a third and a half of patients with depression have treatment-resistant depression (TRD), usually defined as nonresponse to two or more different pharmacological treatments in the current major depressive episode (MDE), taken for an adequate duration and at an adequate dosage [4–7]. These patients have higher relapse rates, poorer long-term clinical and functional outcomes, and substantially lower HRQoL than those who respond to initial treatment [4, 5, 8–11]. Even patients who do achieve clinical remission can experience further declines or only minimal improvements in HRQoL [6].

Specific symptoms of TRD, such as suicidality, anhedonia, insomnia, low energy regardless of sleep, difficulty concentrating, memory issues, and slowed processing speed, have all been reported by patients to particularly reduce their HRQoL [12–17]. Patients have also described difficulties in social functioning, low self-esteem, emotional blunting, and being unable to engage with others, resulting in a detrimental effect on relationships with friends, family, and partners due to an inability to be present emotionally or physically [18]. Treatments that improve self-esteem have been reported as central to providing benefits to HRQoL in patients with TRD [19].

Improvements in these symptoms are not guaranteed with the achievement of clinical outcomes [3, 6, 10]. Treatments that provide not only clinical and functional remission, but also improvements in the lived experience of TRD, therefore, have the best chance of improving the quality of patients’ lives, and these outcomes should be evaluated by clinicians to provide the most comprehensive assessment of treatment efficacy.

Esketamine nasal spray (NS) has demonstrated superior efficacy, including functional and workplace productivity improvements, and a less burdensome safety profile over quetiapine extended release (XR) in patients with TRD, when both were given in combination with an ongoing selective serotonin reuptake inhibitor (SSRI) or serotonin-norepinephrine reuptake inhibitor (SNRI) during the ESCAPE-TRD trial [9, 20, 21]. Additionally, multiple real-world studies have confirmed that esketamine NS leads to significant reductions in depressive symptoms and high rates of clinical response and remission, consistent with those observed in randomized-controlled trials, in patients with TRD in clinical practice [22, 23]. As a result, consensus panels and expert guidance recommendations support esketamine NS as an adjunct to oral antidepressants for TRD after standard pharmacological and augmentation strategies have failed [24, 25].

Here, the effects of esketamine NS on the HRQoL of patients with TRD are reported from a secondary analysis over 32 weeks in ESCAPE-TRD versus quetiapine XR.

A plain language summary of this analysis can be found in the Supplementary Material.

## Methods

### Study design and participants

**Figure 1.**
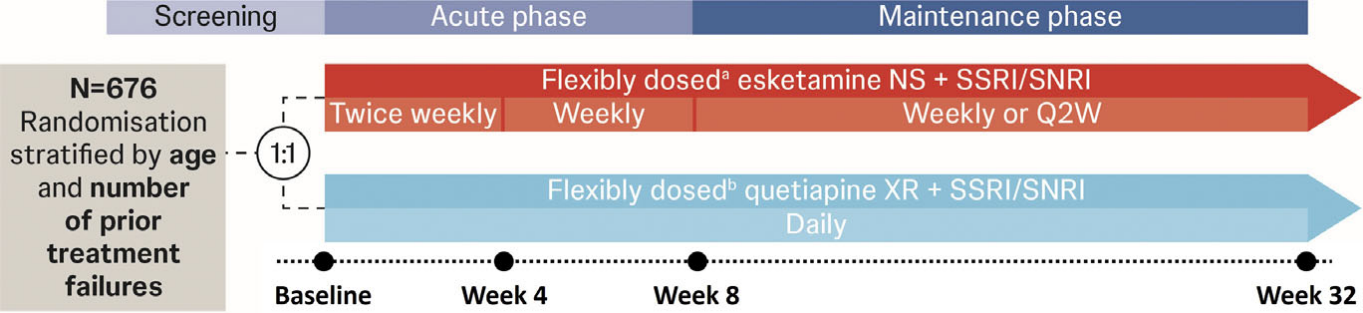
ESCAPE-TRD study design. (A) Esketamine NS was dosed twice weekly (56 mg on Day 1, 56/84 mg from Day 4) from Weeks 1–4, weekly (56/84 mg) from Weeks 5–8, and weekly or Q2W (56/84 mg) from Weeks 9–32, all in addition to an ongoing SSRI/SNRI that elicited nonresponse at baseline. (B) Quetiapine XR was flexibly dosed and administered daily, starting at 50 mg on Days 1–2, 150 mg/day on Days 3–4, and 300 mg/day from Day 5 onwards, all in addition to an ongoing SSRI/SNRI that elicited nonresponse at baseline. NS, nasal spray; Q2W, every 2 weeks; SNRI, serotonin-norepinephrine reuptake inhibitor; SSRI, selective serotonin reuptake inhibitor; XR, extended release.

ESCAPE-TRD (NCT04338321) was a randomized, open-label, rater-blinded, active-controlled phase IIIb study comparing the efficacy and safety of esketamine NS versus quetiapine XR, both alongside an ongoing SSRI/SNRI, in patients with TRD; the full methodology was reported in the primary publication [20]. Patients were randomized 1:1 to esketamine NS or quetiapine XR, both flexibly dosed per label, stratified by age (18–≤64 years; 65–<75 years) and number of prior treatment failures in the current MDE (2 or ≥3) (Figure 1).

ESCAPE-TRD was conducted in accordance with the Declaration of Helsinki [26]. Country-specific ethics review boards provided approval. All patients provided written informed consent, and the study was registered at ClinicalTrials.gov (https://clinicaltrials.gov/study/NCT04338321).

### Patient-reported outcome measures

#### Patient Health Questionnaire-9 (PHQ-9)

The PHQ-9 evaluates patient-reported depressive symptoms using a nine-item questionnaire assessing: anhedonia, low mood, trouble with sleep, fatigue, poor appetite, low self-esteem/guilt, poor concentration, psychomotor agitation/retardation, and thoughts of self-harm. Each item is rated by the patient to indicate how often over the last 2 weeks they have been bothered by the problem, from 0 (*not at all*) to 3 (*nearly every day*), with a total score ranging from 0 to 27; higher scores indicate greater severity of depressive symptoms [27]. The PHQ-9 allows assessment of a patient’s depressive symptoms from their own perspective, which may aid in more effective monitoring of depression when combined with clinician-rated assessments [28].

#### 36-Item Short Form Survey version 2 (SF-36v2)

The SF-36v2 survey measures HRQoL across eight health domains: Physical Functioning, limitations in usual role activities due to physical problems (Role Physical), Bodily Pain, General Health, Vitality, Social Functioning, limitations in usual activities due to emotional problems (Role Emotional), and Mental Health [29]. Questions in each domain assess how much these problems cause limitations in aspects of patients’ lives. Domain scores range from 0 to 100, with higher scores indicating better HRQoL; domain scores were standardized using 2009 US population norms, such that a score of 50 would represent the general population level of HRQoL. The SF-36 is, therefore, useful to assess how far a patient’s HRQoL is from what may be considered “normal” for the general population.

#### Quality of Life in Depression Scale (QLDS)

The QLDS is a 34-item, disease-specific patient-reported outcome measure for assessing the impact of depression on a patient’s HRQoL [30]. Each statement on aspects of patients’ lives related to depression, including, but not limited to, future outlook, self-esteem, self-care, sleep, and enjoyment, is rated 0 (*not true*) or 1 (*true*); total scores range from 0 to 34, with higher scores indicating a lower HRQoL. Patients have confirmed the questions of the QLDS to be relevant to their own experience of depression, indicating its suitability in assessing changes in their HRQoL upon treatment [30].

#### EuroQoL 5-Dimension 5-Level (EQ-5D-5L) and Visual Analogue Scale (EQ-VAS)

The EQ-5D-5L is a generic instrument for describing health based on five dimensions: Mobility, Self-care, Usual Activities, Pain/Discomfort, and Anxiety/Depression. Each dimension has five response levels from no problems (1) to extreme problems/unable to perform the specific domain task (5) [31]. The EQ-VAS records the patient’s self-rated assessment of their overall health status, on a scale of 0 (*worst*) to 100 (*best*) [32]. The five discrete response levels of the EQ-5D-5L allow for greater differentiation between scores, and therefore better sensitivity to changes following treatment, versus scores with fewer response options [33].

### Statistical analysis

Analyses included all randomized patients, using on-treatment visits.

PHQ-9 remission (score ≤ 4) and response (50% improvement from baseline or score ≤ 4) rates, SF-36 domain scores, QLDS change from baseline (CfB) in total score, EQ-5D-5L domain scores of 1 (no problems), and EQ-VAS CfB are reported over time. Time to first PHQ-9 remission or response, as well as time to confirmed remission or response (two consecutive visits), and time to clinically meaningful improvement in QLDS (reduction of ≥8 points) [34] were also estimated.

Proportions of patients reporting PHQ-9 remission and response, clinically meaningful change in QLDS, and “no problems” in each EQ-5D-5L domain are reported alongside the adjusted odds ratios (ORs) and 95% confidence intervals (CIs). Proportions were compared using a Cochran–Mantel–Haenszel chi-square test adjusting for age (18–≤64 years and 65–<75 years) and prior treatment failures (2 and ≥3). Nonresponder imputation (NRI) was applied to treatment discontinuations. For patients who had a missing visit or a missing scale during a visit, but were still receiving study treatment, the missing score was imputed using last observation carried forward (LOCF).

SF-36 domain scores, QLDS, and EQ-VAS total scores were analyzed using a mixed model for repeated measures (MMRM) based on observed cases only (no imputation). The models for QLDS and EQ-VAS included CfB as a dependent variable and baseline score as a covariate, and treatment, age (18–≤64 years and 65–<75 years), prior treatment failures (2 and ≥3), time, and time by treatment as fixed effects, with an unstructured covariance matrix. The model for SF-36 domain score included the score as a dependent variable and age (18–≤64 and 65–<75 years), prior treatment failures (2 and ≥3), time, and time by treatment as fixed effects, with an unstructured covariance matrix. The models were used to estimate least-squares (LS) mean scores and CfB by and between treatment arms along with corresponding 95% CIs.

Time-to-event analyses were conducted using the Kaplan–Meier method. Patients discontinuing study treatment without having reached the event were censored at an infinite (arbitrarily large) time and, hence, were assumed to never achieve the event; patients completing the study (while still on treatment and not having reached the event) were censored at the time of completion. Hazard ratios (HRs) with 95% CIs were estimated using a Cox proportional hazards model, stratified for age (18–≤64 and 65–<75 years) and prior treatment failures (2 and ≥3).

All outcomes reported here were secondary endpoints in ESCAPE-TRD. Consistent with the predefined statistical analysis plan, *p*-values were not adjusted for multiple testing.

## Results

### Patient characteristics and baseline HRQoL

Of 676 total patients, 336 and 340 patients were randomized to esketamine NS and quetiapine XR, respectively. Baseline characteristics, including HRQoL measures, were largely consistent between randomization groups (Supplementary Table 1). Patients had high mean PHQ-9 and mean QLDS scores, low mean SF-36 mental component summary scores, long mean duration of current MDE, and almost half were unemployed, indicating a high burden of TRD on their HRQoL.

### PHQ-9

More esketamine NS-treated patients self-reported no or minimal depressive symptoms by the end of the trial according to the PHQ-9 questionnaire (score ≤ 4), and showed these improvements more quickly on average than quetiapine XR-treated patients.

**Figure 2.**
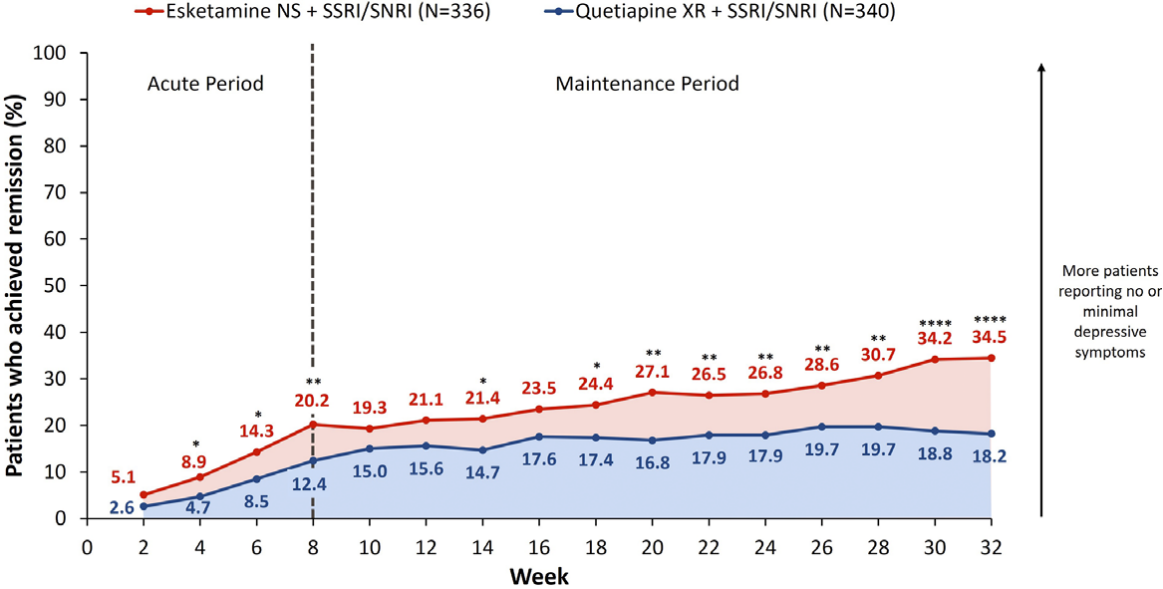
Proportion of patients achieving PHQ-9 remission over time. Full analysis set (includes all randomized patients). NRI was applied to treatment discontinuations. For patients who had a missing visit or a missing scale during a visit, but were still receiving study treatment, the missing score was imputed using LOCF. Tested at a two-sided 0.05 significance level without adjustment for multiple testing. Remission was defined as a PHQ-9 score ≤ 4. *p < 0.05, **p < 0.01, ***p < 0.001, ****p < 0.0001. LOCF, last observation carried forward; NRI, nonresponder imputation; NS, nasal spray; PHQ-9, Patient Health Questionnaire-9; SNRI, serotonin-norepinephrine reuptake inhibitor; SSRI, selective serotonin reuptake inhibitor; XR, extended release.

The percentage of patients achieving PHQ-9-defined remission or response increased over time in both treatment arms. At Week 8, 20.2% of esketamine NS-treated versus 12.4% of quetiapine XR-treated patients achieved PHQ-9-defined remission (OR [95% CI]: 1.80 [1.19, 2.74], *p* = 0.0055), increasing to 34.5% versus 18.2% by Week 32 (OR: 2.39 [1.67, 3.41], *p* < 0.0001, Figure 2). Additionally, 50.0% versus 32.6% of esketamine NS- and quetiapine XR-treated patients were PHQ-9-defined responders at Week 8 (OR: 2.06 [1.51, 2.81], *p* < 0.0001), increasing to 58.0% versus 40.6% by Week 32 (OR: 2.03 [1.50, 2.76], *p* < 0.0001).

Esketamine NS significantly shortened the time to first (Supplementary Figure 1A) and confirmed (Supplementary Figure 1B) PHQ-9 remission versus quetiapine XR (first remission HR [95% CI]: 1.88 [1.50, 2.36], *p* < 0.0001; confirmed remission HR: 1.76 [1.36, 2.29], *p* < 0.0001). Esketamine NS also significantly shortened the time to first and confirmed PHQ-9 response versus quetiapine XR (first response HR: 1.73 [1.44, 2.07], *p* < 0.0001; confirmed response HR: 1.71 [1.41, 2.08], *p* < 0.0001).

### SF-36

**Figure 3.**
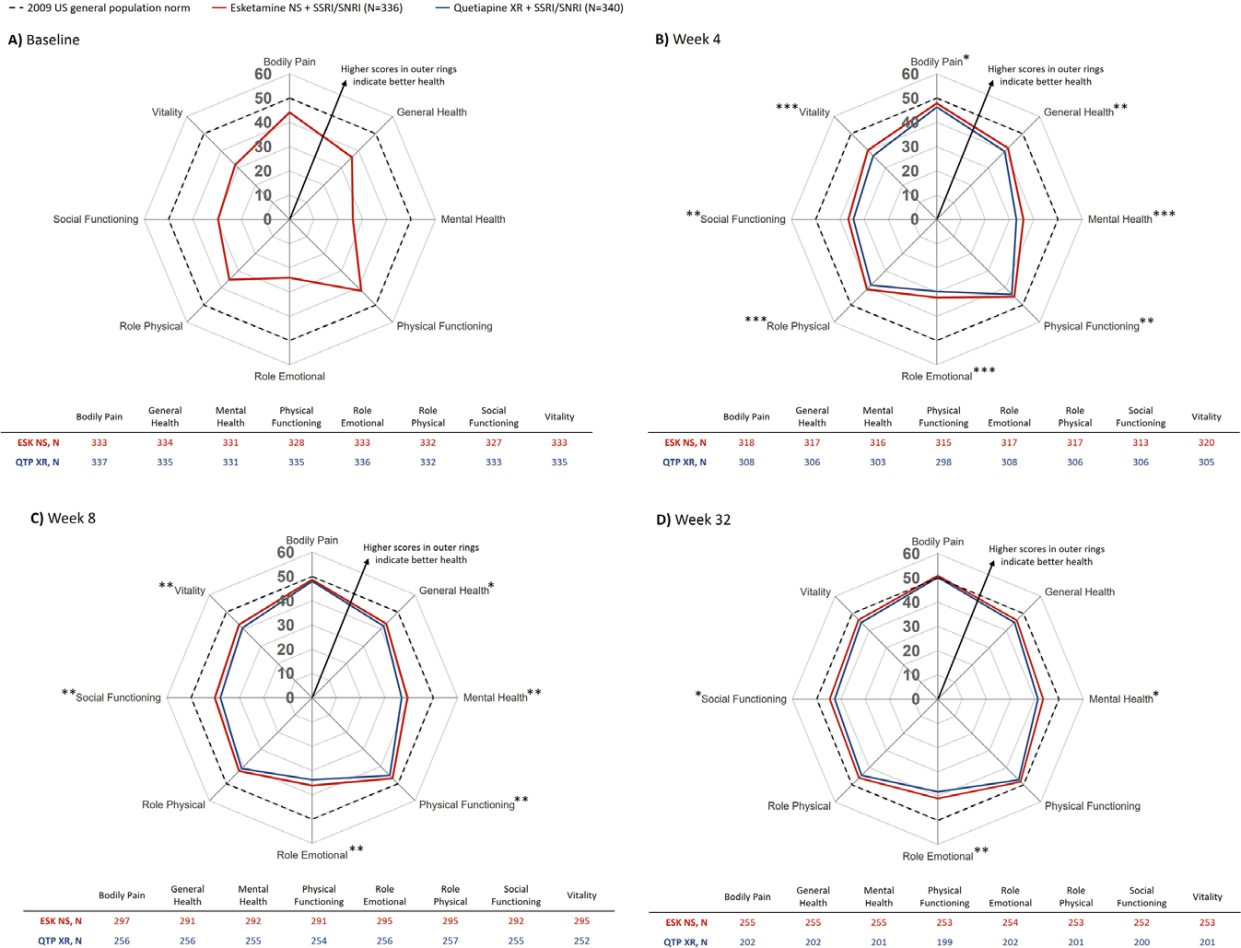
LS mean SF-36v2 domain scores by treatment arm. Full analysis set (includes all randomized patients). Gray dotted lines represent 2009 US population norms. LS means were based on MMRM (based on observed cases; on-treatment visits), adjusted for age and number of prior treatment failures. *p < 0.05, **p < 0.01, ***p < 0.001. ESK, esketamine; LS, least squares; MMRM, mixed model for repeated measures; NS, nasal spray; QTP, quetiapine; SF-36, 36-Item Short Form Survey; SNRI, serotonin-norepinephrine reuptake inhibitor; SSRI, selective serotonin reuptake inhibitor; XR, extended release.

Baseline SF-36v2 domain scores were below what would be considered normal in the general population (Figure 3A), with the lowest scores reported for “Mental Health”, “Role Emotional”, and “Social Functioning”, indicating the greatest burden for patients was in these domains. Improvements in SF-36-measured HRQoL were rapid with esketamine NS and, overall, were larger by the end of the trial than with quetiapine XR.

At Week 4, domain scores were significantly higher with esketamine NS versus quetiapine XR across all domains (Figure 3B). At Week 8, domain scores were significantly higher with esketamine NS versus quetiapine XR across all domains except “Role Physical” and “Bodily Pain” (Figure 3C). By Week 32, most domain scores had returned to levels close to general population norms in both arms (Figure 3D). Domains with the lowest baseline scores showed significantly higher scores with esketamine NS versus quetiapine XR at Week 32: “Role Emotional” (difference [95% CI]: 2.8 [0.8, 4.7], *p* = 0.005), “Mental Health” (difference: 2.1 [0.2, 4.1, *p* = 0.032), and “Social Functioning” (difference: 2.1 [0.4, 3.8], *p* = 0.017); a trend of numerical advantage was seen for all other domains (Figure 3D).

### QLDS

More patients experienced a clinically meaningful improvement in their HRQoL with esketamine NS, and reached this improvement quicker than with quetiapine XR. Esketamine NS-treated patients also had greater overall improvements in QLDS-assessed HRQoL than quetiapine XR-treated patients.

**Figure 4.**
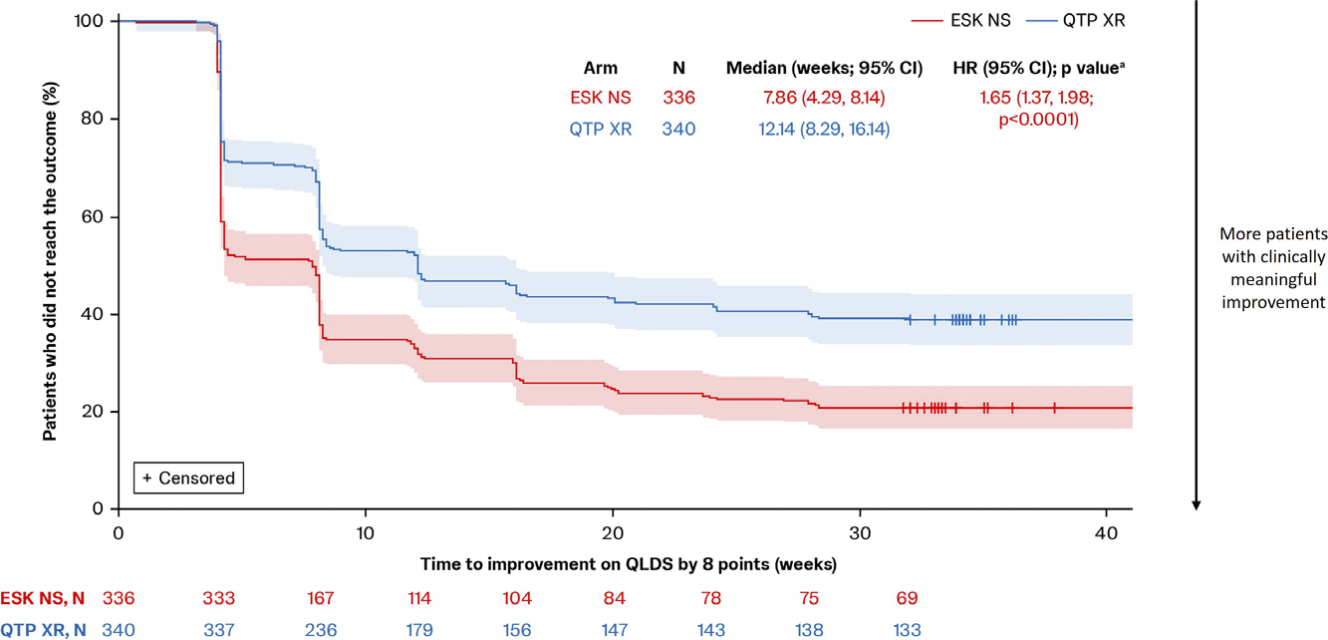
Time to a clinically meaningful improvement in QLDS. Full analysis set (includes all randomized patients). Patients discontinuing treatment were censored at an infinite (arbitrarily large) time and were assumed to never achieve clinically meaningful improvement. Time to first clinically meaningful improvement was defined as the first time a QLDS reduction of ≥8 points was reached. Shaded areas indicate 95% CIs. (A) Tested at a two-sided 0.05 significance level without adjustment for multiple testing. CI, confidence interval; ESK, esketamine; HR, hazard ratio; NS, nasal spray; QLDS, Quality of Life in Depression Scale; QTP, quetiapine; XR, extended release.

A greater proportion of patients treated with esketamine NS achieved a clinically meaningful improvement in QLDS versus quetiapine XR at every time point from Week 4 (48.8% vs. 28.8%; OR [95% CI]: 2.35 [1.71, 3.23]) to Week 32 (60.7% vs. 41.8%; OR: 2.16 [1.59, 2.94]; *p* < 0.0001 at all time points). Esketamine NS also significantly shortened time to meaningful improvement in QLDS versus quetiapine XR (median: 7.86 vs. 12.14 weeks; HR [95% CI]: 1.65 [1.37, 1.98]; *p* < 0.0001; Figure 4).

LS mean CfB in QLDS was significantly greater among patients treated with esketamine NS versus quetiapine XR across all time points through Week 32 (Supplementary Figure 2). At Week 8, LS mean CfB in QLDS with esketamine NS was −11.43 versus –8.61 with quetiapine XR, with a difference of −2.81 (95% CI: −4.23, −1.40; *p* < 0.001). At Week 32, LS mean CfB with esketamine NS was −14.93 versus –12.79 with quetiapine XR, with a difference of −2.14 (−3.69, −0.59; *p* = 0.007).

### EQ-5D-5L and EQ-VAS

Esketamine NS-treated patients showed greater improvements in their overall health state according to the EQ-5D measure than quetiapine XR-treated patients, with more patients indicating that domains most relevant to their condition caused them no problems following treatment.

**Figure 5.**
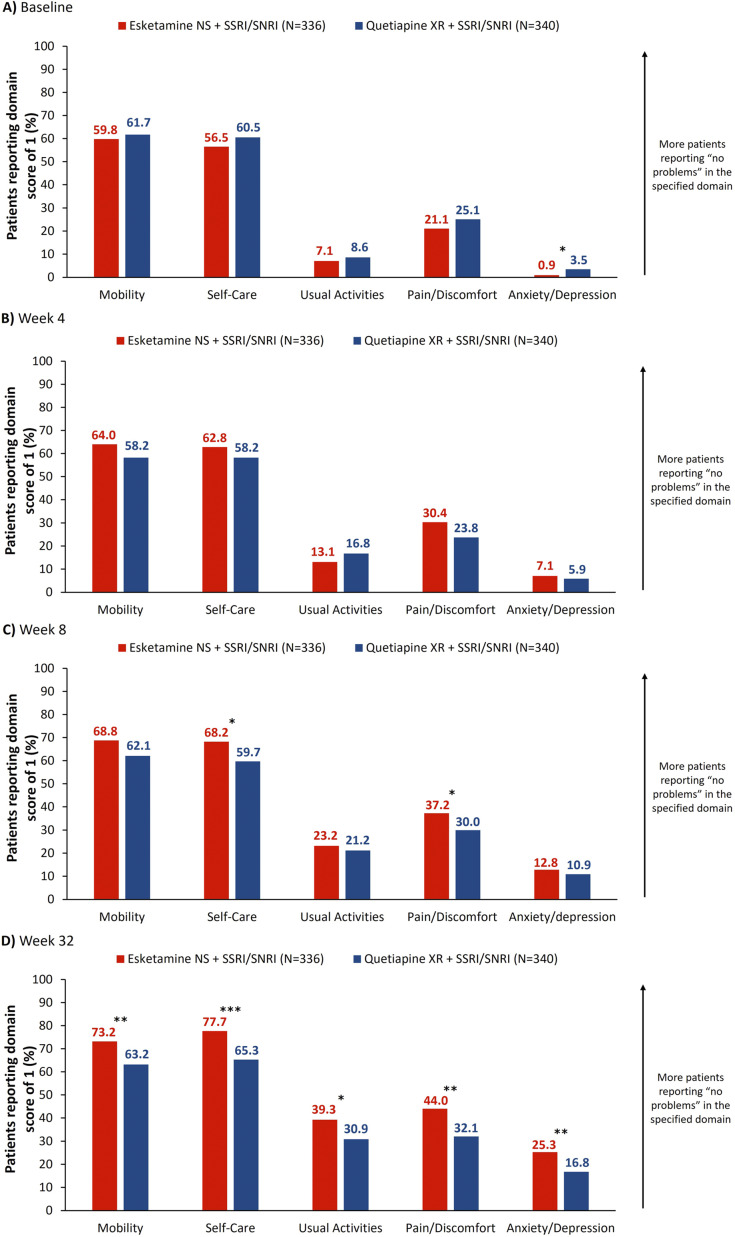
Proportion of patients reporting EQ-5D-5L domain score of 1 (no problems) by treatment arm. Full analysis set (includes all randomized patients). NRI was applied to treatment discontinuations. For patients who had a missing visit or a missing scale during a visit, but were still receiving study treatment, the missing score was imputed using LOCF. *p < 0.05, **p < 0.01, ***p < 0.001, ****p < 0.0001. EQ-5D-5L, EuroQoL 5-Dimension 5-Level; ESK, esketamine; LOCF, last observation carried forward; NRI, nonresponder imputation; NS, nasal spray; QTP, quetiapine; SNRI, serotonin-norepinephrine reuptake inhibitor; SSRI, selective serotonin reuptake inhibitor; XR, extended release.

Proportions of patients reporting an EQ-5D-5L score of 1 (no problems) increased from baseline to Week 32 across all domains (Figure 5A–C). At Week 8, proportions of patients reporting no problems were significantly higher in the “Self-Care” and “Pain/Discomfort” domains: 68.2% and 37.2%, respectively, with esketamine NS and 59.7% (OR [95% CI]: 1.44 [1.05, 1.98], *p* < 0.05) and 30.0% with quetiapine XR (OR: 1.39 [1.01, 1.91], *p* < 0.05; Figure 5B). At Week 32, proportions of patients reporting no problems in these domains were 77.7% and 44.0% with esketamine NS, and 65.3% (OR: 1.85 [1.32, 2.61], *p* < 0.001) and 32.1% with quetiapine XR (OR: 1.68 [1.23, 2.29], *p* < 0.01); differences also reached significance across all other domains at this time (Figure 5C).

At Week 8, LS mean CfB in EQ-VAS with esketamine NS was 19.0 versus 15.0 with quetiapine XR, with a difference of 4.0 (95% CI: 1.2, 6.8; *p* = 0.005; Supplementary Figure 3). At Week 32, LS mean CfB was 24.5 versus 22.2, respectively, with a difference of 2.3 (−0.8, 5.5; *p* = 0.145; Supplementary Figure 3).

## Discussion

Current evidence on the HRQoL burden and subsequent impact of treatment in patients with TRD is largely limited to real-world studies, with a lack of comparison between studies due to variable definitions of TRD [4, 35, 36]. This secondary analysis explored the effects of esketamine NS on aspects of the daily lives of patients with TRD versus quetiapine XR. Esketamine NS provided more rapid and significantly better improvements to HRQoL compared with quetiapine XR across a range of patient-reported measures.

The experience of living with depression has been described in first-person accounts as being unable to experience positive emotions, being trapped in a body drained of energy, and feelings of loneliness or estrangement [37]. Further, patients have self-identified social functioning, interpersonal relationships, and self-confidence as important aspects to evaluate with respect to treatment efficacy [38]. Clinical endpoints, such as remission and response, may therefore only partially reflect patient priorities and, in turn, lead to discordance between clinicians and patients in what may be defined as treatment success [39]. The above-mentioned aspects are therefore crucial to evaluate when measuring treatment efficacy, and they coincide with SF-36 items analyzed here, namely the “Role Emotional”, “Mental Health”, “Vitality”, and “Social Functioning” domains. Results in these domains demonstrated significantly better improvements for esketamine NS-treated patients versus quetiapine XR-treated patients as early as Week 4, with the difference between treatments remaining significant for all except “Vitality” at Week 32. Improvements in “Social Functioning” may mean that patients can reestablish relationships with friends and family members following treatment, while increases in “Vitality” may demonstrate improvements in sleep and energy, aiding in restoring patients’ abilities to perform routine tasks. Improvements in the “Role Emotional” domain may mitigate limitations for patients in social activities due to emotional problems, relieving loneliness and poor self-esteem.

Furthermore, patients with MDD with a delayed response to treatment often experience lower HRQoL compared with those with a rapid response [40]. Treatments that offer a shorter time to improvements in symptoms, and subsequently HRQoL, than current standard-of-care options are therefore warranted [40]. Patients treated with esketamine NS reported significantly better improvements versus quetiapine XR across the SF-36, QLDS, and EQ-5D-5L measures by Week 4, with shorter times to PHQ-9 remission and meaningful improvements in QLDS. These results underline the ability of esketamine NS to provide rapid alleviation of depressive symptoms and improvements in HRQoL, in line with patient preferences. In addition, a return to one’s “usual, normal self and usual level of functioning” has also been identified as an important aspect of treatment [41]. Improvements reported here using the SF-36 measure indicated scores returned to those almost consistent with general population norms in the majority of domains by the end of the trial, while greater proportions of esketamine NS- versus quetiapine XR-treated patients also reported “no problems” across all EQ-5D-5L domains. This provides evidence of not only the speed at which benefits are observed with esketamine NS, but what these benefits mean in the context of patients’ lived experiences.

The similarity of results using patients’ self-reported assessment of their own symptoms (PHQ-9) compared with the clinician-rated outcomes reported in the primary analysis also strengthens the validity of the clinician-rated results. These results, combined with previously reported benefits to patient functioning and work productivity, support the efficacy of esketamine NS beyond the clinical resolution of symptoms [9, 20]. Additionally, recent real-world data have demonstrated the effectiveness of esketamine NS in alleviating anhedonia symptoms [42]. Moreover, the presence of severe anhedonia at baseline has been associated with a more favorable treatment response [43]. It could be suggested that improvements in HRQoL observed here in the esketamine NS group compared with quetiapine XR may, therefore, be mediated by the pro-hedonic effects of esketamine NS. Conversely, the dopaminergic antagonism in patients treated with quetiapine XR may negatively affect reward processing and subjective well-being, which may partly account for differences in HRQoL between treatment arms observed here [44]. However, the effects of both treatments should be taken in the context of the total effect rather than the direct effect to fully capture treatment benefits. It should also be noted that, for some scales, improvements in HRQoL were similar across treatment arms, with room for further improvements remaining after 32 weeks. This indicates that further psychosocial therapy, occupational therapy, other pharmacological interventions, and lifestyle changes, such as a balanced diet, adequate sleep, or regular exercise, may be additional factors to consider to fully normalize HRQoL impairments, underlining the importance of a multidisciplinary approach to care in patients with TRD.

It is well documented that mental health conditions can also translate into physical problems, particularly with chronic disease [45]. Physical health issues, such as weight gain or metabolic syndrome, can arise from treatment with psychiatric medications, or behavioral consequences of the condition itself, and may markedly impact patient HRQoL [46–48]. Furthermore, worsening mental health has been reported as a direct result of physical health issues, thereby creating a reciprocal impact on patients’ HRQoL [49]. The use of several general HRQoL measures here provides a comprehensive evaluation of the impact of TRD on patients’ lives. Significant improvements in the SF-36 “Physical Functioning” domain were seen at Week 4 and Week 8, and in the EQ-5D-5L “Pain/Discomfort” domain at Week 8 and Week 32 versus quetiapine XR, supporting the ability of esketamine NS to alleviate physical discomfort in addition to mental symptoms in TRD, providing improvements to overall patient well-being.

A further aspect of treatment, which may have a significant impact on HRQoL, is the adverse event profile [38]. The safety and tolerability of esketamine NS versus quetiapine XR have been evaluated extensively in ESCAPE-TRD and reported in previous publications [20, 21]. Despite treatment emergent adverse events (TEAEs) being significantly more common with esketamine NS, they led to treatment discontinuation or dosing changes in significantly fewer patients than quetiapine XR, indicative of the comparatively higher burden of events such as weight gain and sedation in quetiapine XR-treated patients; a greater proportion of TEAEs reported with esketamine NS resolved on the same day versus quetiapine XR (92.0% vs. 12.1%). Treatment-emergent suicidal ideation and suicide attempts were seldom reported (esketamine NS: 5 [1.5%] and 2 [0.6%] patients; quetiapine XR: 7 [2.1%] and 1 [0.3%]). The less burdensome tolerability profile of esketamine NS versus quetiapine XR and other commonly prescribed treatments for MDD serves to further support the HRQoL benefits reported in the current analysis [6, 12, 21]. However, it should be noted that the negative impacts of a treatment’s tolerability on patients’ daily lives are likely already reflected to some extent within the patient-reported measures evaluated here.

Given the broad range of aspects identified as important to patients, and the variety of additional factors that may influence individual patient preferences for treatment (e.g., disease severity or personal experiences), taking into account achievement of patients’ personal goals and treatment satisfaction as part of shared decision-making with respect to treatment planning in TRD is, therefore, critical to optimize outcomes, as is advocated in a number of clinical guidelines [50].

Limitations of this analysis include the differing forms of administration for esketamine NS and quetiapine XR, which may have led to expectation bias, as, if a patient had experienced treatment failure in the form of oral medication previously, they may have been more optimistic when taking a different form of medication in esketamine NS versus taking another oral medication. The increased frequency and length of interaction with healthcare personnel, due to the different route of administration and the need for healthcare professional supervision, during administration of esketamine NS versus quetiapine XR, may have also positively influenced patient perceptions surrounding efficacy and led to further improvements in functioning and HRQoL independent of pharmacological treatment. Although, it should be noted that patient-reported outcome measures were assessed before any treatment administration or interaction with healthcare personnel at each visit, and the frequency of clinical interactions in the quetiapine XR group was also greater than the typical frequency in clinical practice, due to the randomized controlled trial framework. Additionally, since in some analyses missing data while on treatment were handled using LOCF, this may have introduced bias by preserving the last observed value and assuming this remained consistent throughout the study, which may not reflect reality; NRI was also applied to treatment discontinuations, and missing at random was applied to MMRM inputs, which may introduce further bias. Finally, while MMRM and time-to-event analyses were adjusted for age and number of prior treatment failures, most analyses were not stratified by additional factors such as sex or oral antidepressant type (SSRI or SNRI). However, such exploratory analyses were conducted, and no meaningful effect of these factors on HRQoL outcomes was identified.

In conclusion, rapid and clinically significant benefits to patients’ daily lives beyond improvements in symptoms of depression were demonstrated with esketamine NS versus quetiapine XR using the SF-36, QLDS, and EQ-5D-5L patient-reported outcome measures. Additionally, measuring patients’ perspectives of their own symptoms using the PHQ-9 assessment showed significantly greater improvements with esketamine NS versus quetiapine XR, in agreement with clinician-rated outcomes from ESCAPE-TRD. These findings demonstrate that esketamine NS treatment in TRD positively impacts aspects of patients’ lives important to them, in parallel to resolving clinical symptoms, which is critical to provide the greatest benefits in routine practice.

## Supporting information

10.1192/j.eurpsy.2025.10123.sm001 Reif et al. supplementary material Reif et al. supplementary material

## Data Availability

The data sharing policy of Johnson & Johnson is available at https://www.jnj.com/innovativemedicine/our-innovation/clinical-trials/transparency. As noted on this site, requests for access to the study data can be submitted through the Yale Open Data Access [YODA] Project site at http://yoda.yale.edu.

## Supplementary material

The supplementary material for this article can be found at http://doi.org/10.1192/j.eurpsy.2025.10123.
