# Supplementary material for: Improvements in health-related quality of life with esketamine nasal spray versus quetiapine extended release
Source: Eur Psychiatry. 2025 Oct 14;68(1):e156. doi: 10.1192/j.eurpsy.2025.10123 (PMC12646121; doi:10.1192/j.eurpsy.2025.10123)
Supplement: Reif et al. supplementary material [file S0924933825101235sup001.pdf]

Improvements in health-related quality of life with esketamine nasal spray versus quetiapine extended release

Andreas Reif,[1,2] Bernhard T. Baune,[3,4] Jozefien Buyze,[5] Anthony J. Cleare,[6] Shaun Johnson,[7] Yerkebulan Kambarov,[5] Nigel Olisa,[7] Falk Schuster,[8] Christian von Holt,[9] Tamara Werner-Kiechle,[9] Eduard Vieta[10]

[1]University Medical Centre Frankfurt, Department of Psychiatry, Psychosomatic Medicine and Psychotherapy, Frankfurt am Main, Germany; [2]Fraunhofer Institute for Translational Medicine and Pharmacology ITMP, Frankfurt am Main, Germany; [3]Department of Psychiatry, University of Münster, Münster, Germany; [4]Department of Psychiatry, The University of Melbourne, Melbourne, Australia; [5]Johnson & Johnson, Beerse, Belgium; [6]Institute of Psychiatry, Psychology & Neuroscience, King's College London, London, UK; [7]GAMIAN-Europe, Brussels, Belgium; [8]Independent consultant, Leipzig, Germany; [9]Johnson & Johnson, Neuss, Germany; [10]Institute of Neuroscience, University of Barcelona, Hospital Clinic, IDIBAPS, CIBERSAM, Barcelona, Spain

SUPPLEMENTARY APPENDIX

Plain Language Summary

Why did we do this study?

Depression can affect a patient's health-related quality of life more than many other long-term illnesses. Patients with treatment-resistant depression (TRD) have not experienced a sufficient improvement in their condition after trying at least two different antidepressant medications. Patients with TRD face difficulties not only with constant low moods, energy and self-esteem, but also with maintaining relationships, employment, studies, hobbies and physical health, which can have a severe impact on the overall quality of their lives. It is important that we understand how new treatments may help to improve the daily lives of people living with TRD, as well as their symptoms of depression.

What did we do?

Esketamine is a drug sprayed into the nose which is used to treat TRD. Here, we compared the effects of esketamine nasal spray versus quetiapine extended release, another medication taken as a tablet, on the health-related quality of life and depression symptoms of patients with TRD. Both drugs were taken alongside a tablet antidepressant. Using a series of patient questionnaires, we explored various aspects of patients' daily lives and the impacts of their depressive symptoms over 32 weeks (8 months).

We looked at the impact of these medications on the symptoms of depression experienced by patients. We did this using the Patient Health Questionnaire-9 (PHQ-9), where the maximum score is 27. A score of 4 or less meant that the patient would have only a low level of depression, or no symptoms of depression at all. If a patient's symptoms halved as compared with before the new medication was started,

it was considered a successful response. We also looked at quality of life using their 36-Item Short Form Survey (SF-36) scores, their Quality of Life in Depression Scale (QLDS) score and their EuroQoL Visual Analogue Scale (EQ-VAS) score. We looked at how these scores changed during the study, and how many patients reported "no problems" in each section of the EuroQoL 5-Dimension 5-Level (EQ-5D-5L) questionnaire. We also looked at the time taken to achieve a big improvement or response using the PHQ-9 and QLDS questionnaires.

What did we find?

The study found that, according to these scales, patients who took esketamine nasal spray experienced bigger improvements in their quality of life and faster relief from depression symptoms compared with those who took quetiapine extended release.

After 32 weeks, more patients on esketamine nasal spray had few or no depression symptoms. Additionally, patients experienced improved emotional wellbeing, energy and ability to socialise, and their quality of life was similar to that of the general public by the end of the study. More patients also reported that they had no problems with looking after themselves and undertaking their usual activities, such as work, study or leisure. Improvements in social life, energy and emotional wellbeing were seen as early as 4 weeks with esketamine nasal spray treatment, which could help patients quickly rebuild relationships, sleep better, feel more energised, and reduce feelings of loneliness and improve self-esteem.

Mental health conditions can also cause physical problems like weight gain, heart issues or organ damage, which can significantly affect a person's quality of life. Improving mental health with treatments like esketamine nasal spray may also help reduce physical problems and improve overall well-being. This was indeed

demonstrated by the results of questions related to physical health issues in the patient-reported scores in this study. Additionally, esketamine nasal spray treatment has fewer and shorter side effects, such as less weight gain and sleepiness, compared with other medications like quetiapine extended release, which may also contribute to improved quality of life in people taking this medication.

Why is this important?

These findings suggest that esketamine nasal spray could offer a more successful option than current treatments for people living with TRD, improving not just their depression symptoms but also their overall quality of life and well-being. Improving patients' quality of life as well as easing depression symptoms is crucial for long-term mental health recovery and everyday life satisfaction.

Supplementary Table 1. Baseline characteristics

Mean (SD), unless otherwise specified	Esketamine NS + SSRI/SNRI N=336	Quetiapine XR + SSRI/SNRI N=340
**Baseline characteristics**		
Age, years	44.3 (13.6)	45.7 (13.4)
Sex, female, n (%)	225 (67.0)	222 (65.3)
Number of treatment failures in the current MDE, n (%)		
2	204 (60.7)	211 (62.1)
≥3	132 (39.3)	129 (37.9)
Employment status, unemployed, n (%)	156 (46.4)	162 (47.6)
**Psychiatric history**		
Age at diagnosis, years	33.5 (11.7)	34.8 (11.7)
Total number of episodes	3.4 (2.4)	3.6 (4.1)
Duration of current episode, weeks	68.8 (84.2)	64.6 (65.7)
Total MADRS baseline score	31.4 (6.1; n=336)	31.0 (5.8; n=339)
PHQ-9 score	17.9 (4.3; n=333)	17.4 (4.5; n=335)
SF-36 scores[a]		
Physical component summary	47.1 (8.9; n=313)	46.9 (9.2; n=312)
Mental component summary	20.5 (7.0; n=313)	21.5 (8.3; n=312)
QLDS score	24.1 (6.5; n=333)	23.6 (6.8; n=334)
EQ-5D-5L VAS score	46.2 (18.1; n=333)	45.7 (17.7; n=338)

Full analysis set: includes all randomised patients. [a] Scores were standardised using 2009 US population norms, so that a score of 50 on any domain would represent the general population level of quality of life. EQ-5D-5L VAS: EuroQoL 5-Dimension 5-Level Visual Analogue Scale; MADRS: Montgomery-Åsberg Depression Rating Scale; MDE: major depressive episode; NS: nasal spray; PHQ-9: Patient Health Questionnaire-9; QLDS: Quality of Life in Depression Scale; SF-36: 36-Item Short Form Questionnaire; SD: standard deviation; SNRI: serotonin-norepinephrine reuptake inhibitor; SSRI: selective serotonin reuptake inhibitor; XR: extended release.

Supplementary Figure 1. Time to PHQ-9 remission

A) Time to first remission

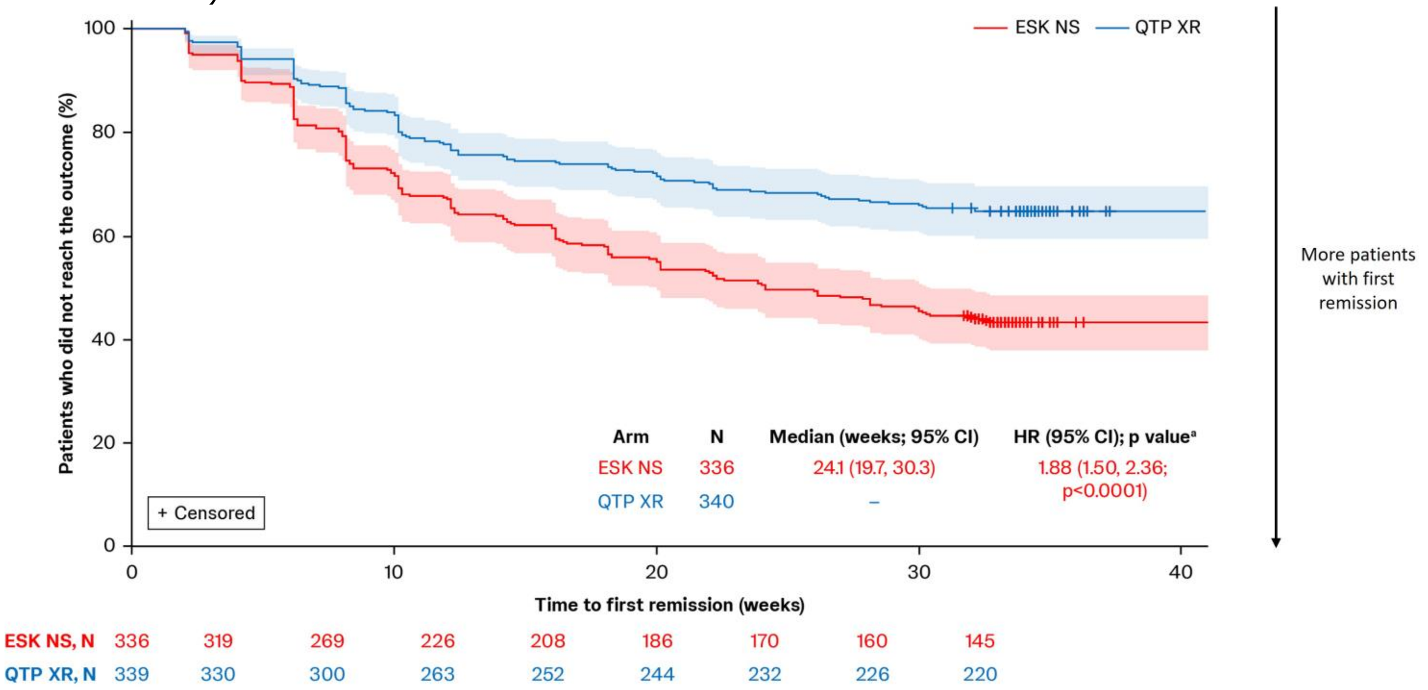

B) Time to confirmed remission

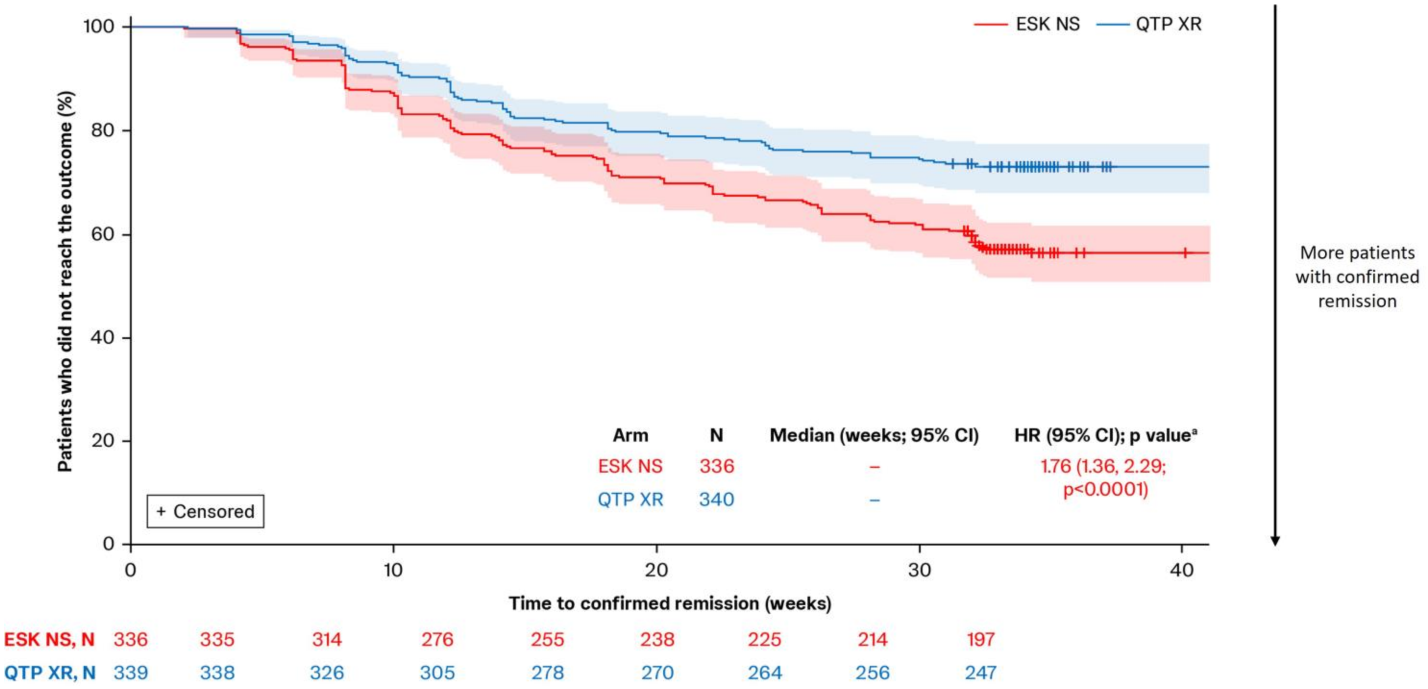

Full analysis set: includes all randomised patients. Patients discontinuing treatment were censored at an infinite (arbitrarily large) time and were assumed to never achieve remission. First remission was defined as a PHQ-9 score ≤4; confirmed remission was defined as a PHQ-9 score ≤4 at two consecutive visits. Shaded area indicates 95% CIs.

[a] Tested at a two-sided 0.05 significance level without adjustment for multiple testing.

CI: confidence interval; ESK: esketamine; HR: hazard ratio; NS: nasal spray; PHQ-9: Patient Health Questionnaire-9; QTP: quetiapine; XR: extended release.

Supplementary Figure 2. Least-squares mean change from baseline in QLDS by treatment arm (MMRM)

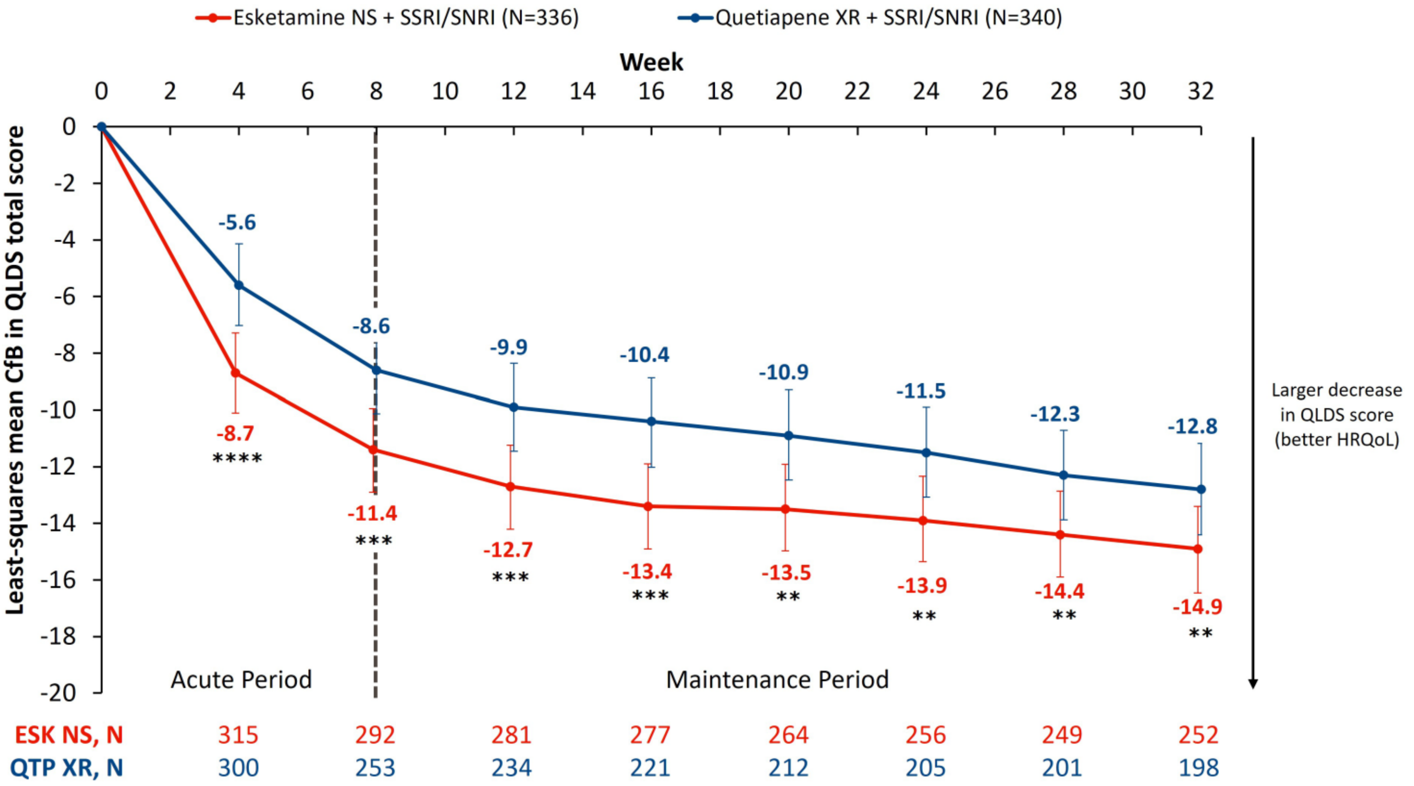

Full analysis set: includes all randomised patients. Error bars depict 95% CIs. Negative CfB refers to a decrease in QLDS score. P values, LS means and 95% CIs were based on MMRM (based on observed cases; on-treatment visits), with baseline score as a covariate, and treatment, age group, number of treatment failures, time and time by treatment as fixed effects. Modelled with an unstructured covariance matrix. Tested at a two-sided 0.05 significance level without adjustment for multiple testing. **p<0.01, ***p<0.001, ****p<0.0001. CfB: change from baseline; CI: confidence interval; ESK: esketamine; HRQoL: health-related quality of life; MMRM: mixed model for repeated measures; NS: nasal spray; QLDS: Quality of Life in Depression Scale; QTP: quetiapine; SNRI: serotonin-norepinephrine reuptake inhibitor; SSRI: selective serotonin reuptake inhibitor; XR: extended release.

Supplementary Figure 3. Least-squares mean change from baseline in EQ-VAS by treatment arm (MMRM)

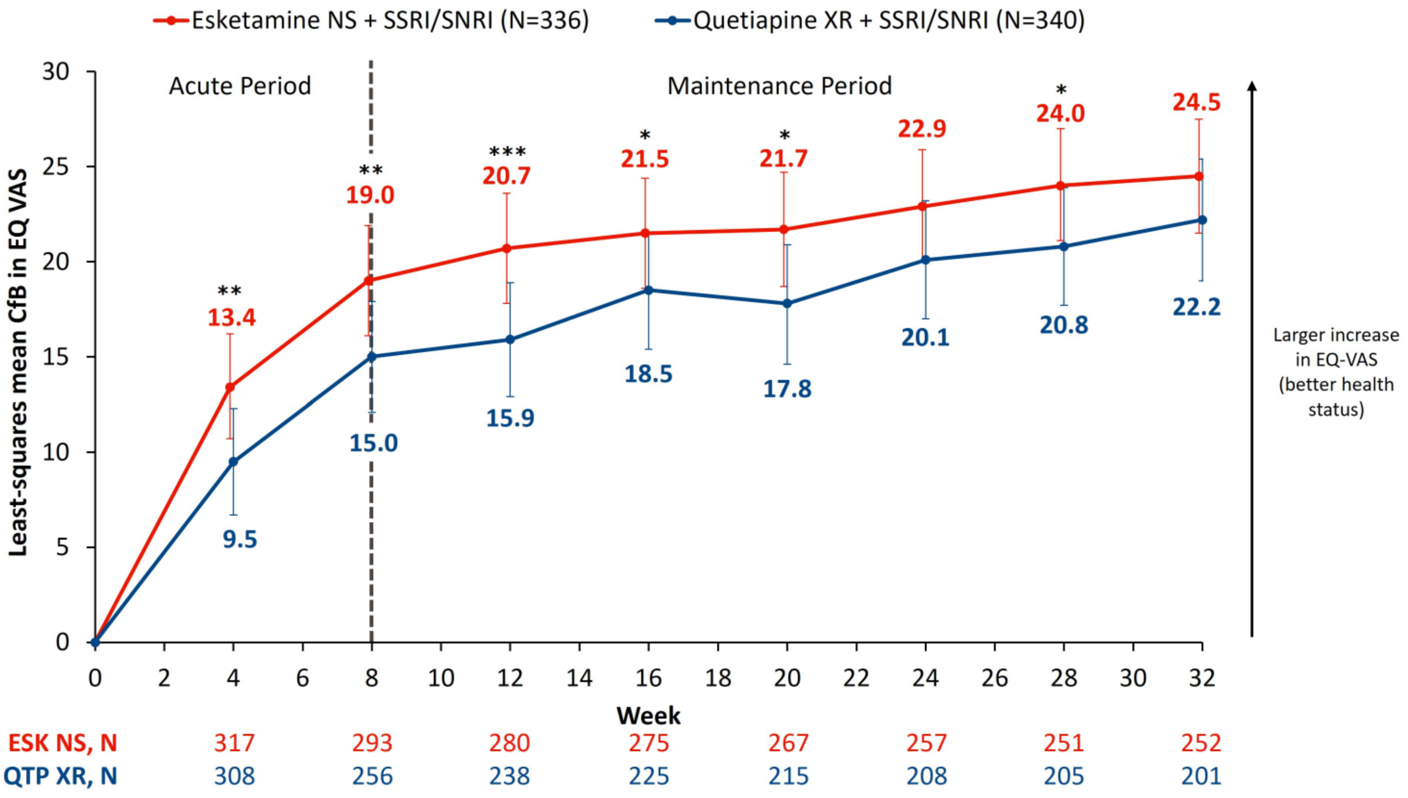

Full analysis set: includes all randomised patients. Error bars depict 95% CIs. Positive CfB refers to an increase in EQ-VAS score. P values, LS means and 95% CIs were based on MMRM (based on observed cases; on-treatment visits), with baseline score as a covariate, and treatment, age group, number of treatment failures, time and time by treatment as fixed effects. Modelled with an unstructured covariance matrix. Tested at a two-sided 0.05 significance level without adjustment for multiple testing. *p<0.05, **p<0.01, ***p<0.001. CfB: change from baseline; ESK: esketamine; EQ-VAS: EuroQoL Visual Analogue Scale; MMRM: mixed model for repeated measures; NS: nasal spray; QTP: quetiapine; XR: extended release.
